# Draft genome of a human gut-derived *Blautia* sp. that ameliorates colitis and colitis-associated sociability deficits in mice

**DOI:** 10.1128/mra.00726-24

**Published:** 2024-11-29

**Authors:** Michaela A. Murphy, D. Garrett Brown, Rickesha S. Bell, Allison M. Weis, Logan A. Barrios, W. Zac Stephens, June L. Round

**Affiliations:** 1 Department of Pathology, Division of Microbiology and Immunology, Huntsman Cancer Institute, University of Utah School of Medicine, Salt Lake City, Utah, USA; Montana State University, Bozeman, Montana, USA

**Keywords:** *Blautia*, microbiota, colitis

## Abstract

*Blautia* is a genus of anaerobic, gram-positive bacteria commonly found in mammalian gastrointestinal tracts. Yet, how variations among different *Blautia* strains can impact host health is poorly understood. We present a *Blautia* sp. genome isolated from human feces whose supplementation to mice can ameliorate colitis severity and associated sociability deficits.

## ANNOUNCEMENT

Conflicting evidence exists regarding the influence of *Blautia* on human disease (1, 2). For instance**,** *Blautia* is reduced in gut microbiotas of Crohn’s disease and colorectal cancer patients (3, 4), and work from our group showed supplementation of this *Blautia* isolate can reduce colitis severity and colitis-associated sociability deficits in mice (5). However, other studies correlate *Blautia* abundance with irritable bowel syndrome and ulcerative colitis (6–8). As species-level distinctions were not made in these analyses, further investigation into this genus is warranted to understand its impact on human health.

*Blautia* sp. JLR.GB0024 was isolated from feces of ex-germfree C57BL/6J mice, reared in-house, that had undergone a human-to-mouse fecal microbial transplant in the Round lab under University of Utah IACUC protocol #20-03006 (9). The human sample was from a neurotypical subject in the Genetics of Autism study, approved by the University of Utah IRB #00006042. Feces from these mice were plated on gut microbiota medium and incubated for 7 days at 37°C anaerobically (9). Colonies were diluted in tryptone yeast extract glucose medium to obtain a collection of individual bacterial cells (9). After incubation (37°C, 5 days), isolates were preserved, and DNA was extracted with a Purelink Microbiome DNA purification kit (Invitrogen) (9). JLR.GB0024 was identified as *Blautia* by Sanger 16S rRNA gene sequencing using primers 16S_F:*AGAGTTTGATC*MTGGC and 16S_R:*TACCTTGTTACGACTT* and the Silva classifier (10).

Sequencing was performed with an Illumina NovaSeq with paired-end 150-cycle sequencing and Oxford Nanopore Technologies (ONT) minION with a flongle flowcell (version FLO-FLG114). Illumina libraries were prepared with the NEBNext Ultra II FS DNA kit (NEB, E7805S). Reads were quality and adapter-filtered with cutadapt (v2.10) (11) in the Trim Galore (v0.6.6) wrapper. ONT libraries were prepared without additional DNA shearing or size selection using a rapid barcoding kit with R10.4.1 chemistry (ONT, SQK-RBK114-24). ONT reads were basecalled and adapter-trimmed with guppy (v6.4.2_gpu) resulting in 1,837 long reads (6.3 MB; 6,381 N_50_) that were quality-filtered with NanoFilt (12) for minimum average read-quality 10 and length 200.

The genome was hybrid assembled using SPAdes v3.15.5 within Unicycler v0.5.0 (“normal mode,” default parameters except only contigs >200 bp were kept) (13, 14) using all ONT reads and Illumina reads downsampled to 4M paired-end reads (sequence depth of ~197×). The assembly was annotated using GenBank PGAP v6.6 (15). The assembled genome contains 6,068,419 bps**,** comprising 5,492 predicted genes and 46.5% GC content. It has 66 contigs with an N_50_ value of 348.9 Kb. CheckM v1.2.2 revealed completeness of 98.3% (16).

The NCBI taxonomy of JLR.GB0024 is Bacillota phylum, Clostridia class, Eubacteriales order, Lachnospiraceae family, and *Blautia* genus. The nearest reference genome is *Blautia parvula* sp. nov. NBRC113351 GCA_020215665.1, by 98.84% sequence identity [FastANI run within GTDB-tk (version 2.3.0)] (17). The JLR.GB0024 genome contains putative genes involved in butyrate (*buk*), tryptophan (*trpB, trpC, aroC*), and vitamin (*cbiB, bioB*) production (Table 1). It also contains tetracycline resistance components (*tet(W*)) and CRISPR machinery (*cas1c*) (Table 1).

**TABLE 1:** Key *Blautia* sp. JLR.GB0024 genomic elements

Gene	Protein ID	Type	Protein function
*tet(W*)	MEI1258411.1	AMR	Tetracycline resistance ribosomal protection protein
*cas1c*	MEI1255721.1	CRISPR	Type I-C CRISPR-associated endonuclease
*aroB*	MEI1253864.1	Metabolism	3-dehydroquinate synthase
*aroC*	MEI1255768.1	Metabolism	Chorismate synthase
*bglX*	MEI1257670.1/MEI1257799.1	Metabolism	Beta-glucosidase
*bioB*	MEI1256778.1	Metabolism	Biotin synthase
*buk*	MEI1255779.1	Metabolism	Butyrate kinase
*cbiB*	MEI1256276.1	Metabolism	Adenosylcobinamide-phosphate synthase
*fdhF*	MEI1256074.1	Metabolism	Formate dehydrogenase subunit alpha
*gdhA*	MEI1255098.1	Metabolism	NADP-specific glutamate dehydrogenase
*gltA*	MEI1253742.1	Metabolism	NADPH-dependent glutamate synthase
*iorA*	MEI1257288.1	Metabolism	Indole pyruvate ferredoxin oxidoreductase subunit alpha
*trpB*	MEI1256337.1	Metabolism	Tryptophan synthase subunit beta
*trpC*	MEI1256335.1	Metabolism	Indole-3-glycerol phosphate synthase
*unnamed*	MEI1253999.1/MEI1258136.1	Metabolism	Glutamine synthetase III
*terL*	MEI1255994.1	Prophage	Phage terminase large subunit
*unnamed*	MEI1254010.1	Secretion	Type II secretion system F family protein
*yabQ*	MEI1255632.1	Sporulation	Spore cortex biosynthesis protein

## Data Availability

The genome assembly and reads are available under accession numbers JBANBF000000000.1, SRR28100816 (Illumina), and SRR28100815 (ONT). The isolate is available from the Round lab.
